# Neonatal Mortality Due to Early-Onset Sepsis in Eastern Europe: A Review of Current Monitoring Protocols During Pregnancy and Maternal Demographics in Eastern Europe, with an Emphasis on Romania—Comparison with Data Extracted from a Secondary Center in Southern Romania

**DOI:** 10.3390/children12030354

**Published:** 2025-03-13

**Authors:** Anca Vulcănescu, Mirela-Anișoara Siminel, Sorin-Nicolae Dinescu, Anda-Lorena Dijmărescu, Maria-Magdalena Manolea, Sidonia-Maria Săndulescu

**Affiliations:** 1 “Filantropia” Clinical Municipal Hospital, 200143 Craiova, Romania; anca.vulcanescu@umfcv.ro (A.V.); lorena.dijmarescu@umfcv.ro (A.-L.D.); magdalena.manolea@umfcv.ro (M.-M.M.); 2 University of Medicine and Pharmacy of Craiova, 200349 Craiova, Romania; 3 Department of Neonatology, University of Medicine and Pharmacy of Craiova, 200349 Craiova, Romania; 4 Department of Epidemiology, University of Medicine and Pharmacy of Craiova, 200349 Craiova, Romania; 5 Clinical Emergency County Hospital, 200642 Craiova, Romania; 6 Department of Obstetrics and Gynecology, University of Medicine and Pharmacy of Craiova, 200349 Craiova, Romania

**Keywords:** neonatal mortality, maternal demographics, monitoring protocols, Eastern Europe

## Abstract

Neonatal mortality, particularly due to early-onset sepsis (EOS), remains a pressing issue in Eastern Europe, with Romania experiencing one of the highest neonatal mortality rates in the European Union. EOS, caused by bacterial infections within the first 72 h of life, significantly contributes to neonatal deaths, particularly in rural and underserved areas where healthcare resources are limited. Disparities in prenatal care access, maternal demographics, and systemic healthcare gaps worsen this issue, highlighting the need for comprehensive interventions. Objectives: This review aims to examine the prevalence of neonatal mortality due to EOS in Romania and the broader Eastern European context, focusing on current prenatal care monitoring protocols and maternal demographics, and comparing the available literature with new data. By evaluating disparities in healthcare access, costs, and outcomes, this study identifies systemic barriers to maternal and neonatal care. Methods: A systematic review of 100 articles was conducted using PRISMA guidelines. Key sources included PubMed, Google Scholar, and open-access journals, with 25 articles meeting the inclusion criteria. The analysis encompassed maternal age, parity, literacy levels, marital status, and their impact on neonatal outcomes, alongside the financial burden of prenatal care. Results: The findings underscore stark inequalities in healthcare delivery between rural and urban regions, where limited prenatal visits, out-of-pocket costs, and cultural barriers hinder prompt EOS prevention and management. Advanced maternal age, low literacy, and socioeconomic disparities were identified as critical risk factors. Conclusions: To reduce neonatal mortality due to EOS, targeted efforts must address healthcare accessibility, improve prenatal care protocols, and integrate culturally sensitive practices. These interventions can bridge systemic gaps and promote equitable health outcomes for mothers and newborns across Eastern Europe.

## 1. Introduction

### 1.1. Current Data on Neonatal Mortality and Early-Onset Sepsis (EOS) in Eastern Europe

Neonatal mortality rates (NMRs) in Eastern Europe remain higher than those in Western Europe, with early-onset sepsis (EOS) being a significant contributor to neonatal deaths. According to the World Health Organization (WHO) and UNICEF (United Nations International Children’s Emergency Fund), neonatal deaths in the first 28 days of life account for a substantial proportion of overall infant mortality, with infections such as EOS being a major factor in this burden [1,2]. In Romania, the neonatal mortality rate is approximately 4.7 deaths per 1000 live births, which, while showing some improvement over the past decade, remains one of the highest in the European Union [3]. EOS is a critical contributor to these deaths, often due to delayed diagnosis, inadequate neonatal care, and insufficient access to timely medical interventions in underserved areas [4].

Data from Eurostat (the European Union’s statistical office, providing reliable data on economics, demographics, trade, and social issues for policymaking and public use) highlight a persistent disparity in neonatal outcomes between Eastern and Western Europe, driven by systemic differences in healthcare access, socioeconomic inequalities, and quality of prenatal care [3]. For example, while countries in Western Europe have achieved neonatal mortality rates of approximately 2 deaths per 1000 live births, Romania and other Eastern European nations continue to struggle with higher rates due to preventable neonatal infections, including EOS. Despite improvements in healthcare infrastructure, Romania still lags behind Western European standards, reflecting gaps in healthcare delivery and the prevalence of infections conditions like EOS, particularly in rural and marginalized communities [5,6].

### 1.2. Trends in Neonatal Mortality over the Past Decade

Over the last decade, Eastern Europe has seen a gradual decline in neonatal mortality rates, thanks to increased health interventions and awareness programs. For instance, neonatal mortality in Romania decreased from 8 per 1000 live births in 2010 to approximately 4.7 per 1000 live births in 2020 [3]. This improvement can be attributed to global initiatives such as the WHO’s Every Newborn Action Plan and UNICEF’s maternal and child health programs, which have focused on reducing neonatal mortality through better access to healthcare services and improved prenatal and neonatal care [2].

However, this decline has been uneven, with rural and underserved areas experiencing slower progress compared to urban centers. Studies indicate that while urban areas in Romania have benefited from improved healthcare infrastructure and access to advanced diagnostic tools, rural regions continue to face challenges such as limited healthcare facilities, shortages of trained personnel, and delayed interventions [7]. These disparities exacerbate the burden of EOS-related mortality in rural populations, where timely diagnosis and treatment are often unavailable [8].

Additionally, socioeconomic factors play a critical role in neonatal outcomes. Families in rural areas or from marginalized communities, such as the Roma population, face significant barriers to accessing prenatal and neonatal care due to financial constraints and systemic inequities [5]. This highlights the urgent need for targeted interventions to address healthcare disparities and ensure fair access to essential services across all regions of Romania.

Maternal health monitoring during pregnancy is integral to reducing complications and improving perinatal outcomes. Across Europe, prenatal care protocols are set up by using guidelines from the World Health Organization (WHO) and local health ministries. However, significant regional variations exist in the implementation of these protocols, particularly in Eastern Europe. Romania, a country with notable disparities in healthcare access, provides a case study to examine the interplay between maternal demographics and prenatal monitoring.

This review focuses on current pregnancy monitoring protocols in Europe, with an emphasis on Romania, maternal demographic factors (such as age, parity, marital status, and literacy), and the costs and burdens associated with prenatal care. These maternal characteristics are closely linked to neonatal vulnerabilities. For instance, maternal age is associated with neonatal risks, with advanced age or adolescence contributing to complications such as preterm birth and low birth weight, which are significant risk factors for EOS. Similarly, parity influences care-seeking behavior and pregnancy-related complications, while marital status and literacy often reflect levels of socioeconomic support and health literacy, both of which impact healthcare access and adherence to medical advice. In particular, maternal education plays a vital role in recognizing neonatal distress signals, such as those associated with EOS, and ensuring timely medical interventions.

Additionally, examining the financial and logistical burdens of prenatal care is essential in understanding systemic barriers that can affect maternal compliance and access to monitoring protocols, especially in resource-limited settings like Romania. Limited access to quality and affordable healthcare, particularly in rural areas, may delay the identification and management of maternal infections that contribute directly to neonatal EOS. While much is known about neonatal mortality and EOS, this review addresses an existing gap in the literature by focusing on how these maternal and systemic factors interact. In doing so, it highlights key challenges and provides insights for improved prenatal care and reduced neonatal mortality.

## 2. Materials and Methods

### 2.1. Search Strategy

A systematic review was conducted following PRISMA guidelines (Supplementary Material S1) to ensure a comprehensive evaluation of the literature, for articles published between January 2000 and August 2024. Searches were performed by two of the authors (A.V., M.S.) between August and September 2024, across PubMed, Google Scholar, and open-access journals using targeted keywords such as “pregnancy monitoring protocols Europe”, “maternal demographics Romania”, “neonatal mortality Eastern Europe”, and “early-onset sepsis.” Boolean operators (e.g., AND, OR) were employed to refine results and interrogate databases thoroughly. For example, an extended search query in PubMed was constructed using the following sequence: (“neonatal mortality” OR “early-onset sepsis” OR “EOS”) AND (“prenatal care” OR “pregnancy monitoring”) AND (“Eastern Europe” OR “Romania”) AND (“maternal demographics” OR “maternal age” OR “parity”) AND (“health disparities” OR “rural healthcare”).

**Figure 1:**
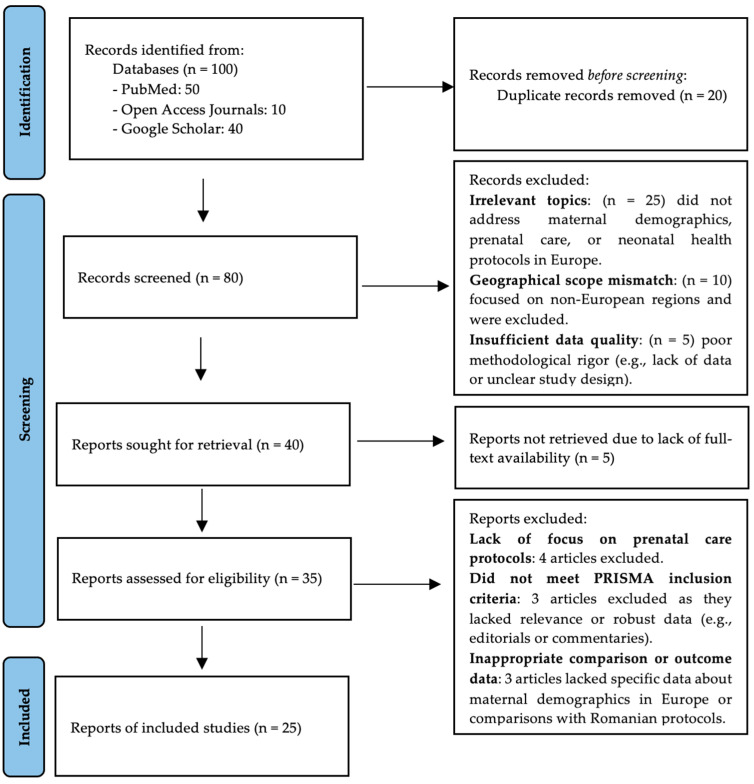
Prisma flow chart.

Literature reports from organizations like the WHO and UNICEF and references from retrieved articles were also screened. After identification, articles were imported into reference management software for organization and duplicate removal, as it can be seen in Figure 1. A detailed search strategy can be found in Supplementary Material S3.

### 2.2. Inclusion and Exclusion Criteria

Articles were included if they met the following criteria:

- Examined neonatal mortality, EOS, or maternal health in Eastern Europe.
- Addressed prenatal monitoring protocols or maternal demographics.
- Were peer-reviewed, published between January 2000 and August 2024, and written in English.

Exclusion criteria included the following:

- Studies lacking full text.
- Non-research articles (e.g., opinion pieces, editorials).
- Papers focused on non-human subjects or regions outside Europe.

### 2.3. Data Extraction

A standardized data extraction form was developed to collect information on study characteristics (e.g., author, year, location), population demographics, prenatal care protocols, outcomes related to EOS, and statistical findings. Extracted data were cross-verified by two reviewers for accuracy (A.V., M.S.). When data were unclear, assumptions were made based on the available study context, and no contact with study investigators was made for missing data.

Risk of bias assessment was performed by two of the authors (A.V., M.S.), and it was found that most of the studies (82%) were robust and had minimal bias, making them high-quality studies. The categories considered were study design, sample size, outcome reporting, funding bias, and conflicts of interest. A detailed assessment can be found in Supplementary Material S2.

Descriptive analyses summarized key study characteristics, including maternal age, parity, literacy, and geographic distribution (e.g., rural vs. urban). Additionally, subgroup analyses explored disparities between rural and urban populations, highlighting socioeconomic and geographic factors influencing neonatal outcomes.

Regarding the statistical software, we used Microsoft Excel (Version 16.93.1 (25011917)) and macOS numbers (version 14.3 (7042.0.76)) (used to organize raw data and generate simple summary statistics). No specialized bias assessment software was directly used.

### 2.4. Study Design

The data we compare to the European and Romanian trends resulted from a study conducted in a secondary neonatology center in southern Romania, which took place over a period of 6 months (April 2024–September 2024), as part of a doctoral thesis, focusing on the epidemiologic aspects of early-onset sepsis. Data regarding 442 newborns and their mothers were extracted during this interval, analyzed, interpreted, and compared to the existing data in the literature, aiming to improve our current practice regarding suspected EOS cases, with one of our main goals being to reduce antibiotic use in newborns. In this article, we chose to discuss the demographic data concerning the mothers included in our study, comparing our findings to the data already available.

### 2.5. Results

To ensure the focus and relevance of this review, we excluded studies that did not align with the study’s objectives or geographic scope. For instance, Alemán Riganti (2024) [9] examined prenatal monitoring strategies and neonatal mortality in Latin America, a region outside the Eastern European focus of this analysis. Similarly, Aniekwu and Uzodike (2008) [10] reviewed reproductive health law and policy reforms in the African sub-region. These exclusions ensured that the included studies offered direct insights into maternal risk factors, prenatal monitoring protocols, and socioeconomic disparities affecting neonatal outcomes in Eastern Europe.

The limitations identified after assessing the risk of bias included small sample sizes in studies focusing on rural populations and marginalized groups selective outcome reporting in studies prioritizing specific neonatal or maternal outcomes; and few studies disclosing funding sources, raising concerns about potential funding or resource biases. Future studies should adopt randomized or comparative designs, increase sample sizes for generalizability, and report funding sources transparently. Regarding the heterogeneity assessment, significant variations between studies due to differences in healthcare access and socioeconomic factors were noted. The review findings can be found in Table 1 and Table 2.

## 3. Review Findings

### 3.1. Current Monitoring Protocols During Pregnancy in Europe

#### 3.1.1. Standardized Guidelines

WHO guidelines recommend a minimum of eight antenatal visits for low-risk pregnancies, including regular ultrasounds and clinical assessments. The European Union has largely adopted these recommendations, but implementation varies significantly by country [29]. In high-income countries such as Germany and France, adherence to the recommended protocol is nearly universal. In Romania, limited resources in rural areas mean that many women attend fewer visits than recommended, often only 4–5 visits, especially when healthcare facilities are distant or access to healthcare professionals is restricted [23].

#### 3.1.2. Prenatal Monitoring

Western Europe: Comprehensive prenatal care includes 8–12 visits, with screenings for infections like Group B Streptococcus (GBS) [30,31].

In contrast, Romania and other Eastern European nations face systemic challenges. Western European countries, such as the UK and France, integrate advanced technologies and multidisciplinary approaches into prenatal care. Eastern Europe lags in implementing uniform monitoring practices, partly due to resource constraints. For instance, while GBS screening is routine in Western Europe, it is inconsistently performed in Romania due to cost [17].

Prenatal monitoring in Eastern Europe generally encompasses essential components such as routine ultrasounds, blood testing, urine analyses, and screenings for conditions like gestational diabetes and various infections. However, the extent and frequency of these monitoring protocols differ considerably among countries and across different regions [8].

Fewer visits (4–8) and limited infection screenings are reported, particularly in rural areas [14,21]. At the same time, the maternal cost burden remains significant in Eastern Europe, with out-of-pocket expenses for essential tests (e.g., GBS, TORCH infections) [21,32].

In Romania, while the public healthcare system offers basic prenatal services, families often find themselves needing to pay out of pocket for additional diagnostics, including advanced ultrasounds and genetic screenings, which can create barriers to comprehensive care [33].

#### 3.1.3. Romanian Prenatal Care Protocol

In Romania, the Ministry of Health recommends the following:

Minimum number of visits: At least eight visits for uncomplicated pregnancies, advocating for monthly obstetric visits until the 28th week, transitioning to bi-weekly visits until the 36th week, followed by weekly visits until delivery. 

Ultrasounds: Three key ultrasounds are recommended: the first ultrasound aims to confirm the pregnancy and estimate gestational age (first trimester), while the second one, usually at 20–24 weeks, screens for structural abnormalities (second trimester). The third ultrasound assesses fetal growth, position, and wellbeing (third trimester).

Blood and bacteriological tests: The typical protocol for blood testing in Romania includes initial blood typing, screening for infectious diseases (HIV, syphilis, and hepatitis B), and assessing for anemia. Additionally, bacteriological screenings, such as Group B Streptococcus (GBS) testing, are recommended in the third trimester [12]. While these tests are included in standard care, out-of-pocket costs often influence compliance, particularly among lower-income populations [16,32].

For instance, some women may skip GBS screening if their local clinic does not provide it or if they cannot afford additional testing fees, which typically range from EUR 10 to EUR 50 per test [34].

While public healthcare covers basic tests such as blood and urine tests, many additional screenings require out-of-pocket payments. 

First-Trimester Screening: Combined screening for Down syndrome (nuchal translucency ultrasound and maternal blood tests) is not universally covered, and many families must pay privately for these tests.

Infections Screening: While syphilis, HIV, and hepatitis B tests are included in public care, other important screenings, such as for Group B Streptococcus (GBS), are not routinely performed, increasing the risk of neonatal infections like early-onset sepsis (EOS) [35].

Gestational Diabetes and Preeclampsia Screening: Screening for gestational diabetes and preeclampsia is part of the standard prenatal care package, but challenges remain in ensuring universal coverage. In rural areas, many women are not screened due to lack of equipment, trained staff, or limited awareness of the importance of these tests.

#### 3.1.4. Advanced Diagnostics

Advanced diagnostics, such as genetic testing (e.g., non-invasive prenatal testing or NIPT) and 3D/4D ultrasounds, are available primarily through private healthcare providers. These services are prohibitively expensive for many families, with costs ranging from EUR 500 to 1500 per pregnancy, creating a significant financial burden for low-income households [25].

However, compliance with these guidelines is inconsistent, particularly in rural regions. A report by Miteniece et al. (2021) indicates that only 50% of pregnant women in rural Romania adhere to the full monitoring protocol due to logistical and financial barriers [15].

### 3.2. Maternal Demographics in Eastern Europe and Their Impact

Maternal demographics in Eastern Europe are shaped by significant socioeconomic disparities, with rural and marginalized populations facing the greatest challenges. In Romania, approximately 46% of the population lives in rural areas, where access to healthcare is limited [3]. Marginalized groups, such as the Roma population, are disproportionately affected, with higher rates of poverty, lower educational attainment, and limited access to prenatal care [5].

Studies show that younger maternal age, lower educational levels, and lower socioeconomic status are associated with poorer pregnancy outcomes in Eastern Europe. For example, in Romania, teenage pregnancies are more common in rural areas, where access to contraception and reproductive health education is limited. These factors contribute to higher rates of maternal and neonatal complications, including preterm births and infections [3,4].

#### 3.2.1. Maternal Age

Maternal age significantly influences prenatal care uptake and outcomes. Younger maternal age correlates with limited health literacy and reduced prenatal care compliance. Conversely, advanced maternal age (>35 years) in Romania is associated with increased obstetric complications but better care adherence in urban areas.

Eastern Europe Trends

Maternal age is a crucial determinant of pregnancy outcomes, with both advanced maternal age (≥35 years) and adolescent pregnancies (<20 years) posing increased risks for complications. In Eastern Europe, the average maternal age at childbirth ranges between 27 and 30 years, influenced by socioeconomic status and access to contraception and education. Teen pregnancies are particularly prevalent in rural areas where access to reproductive education and family planning services is limited [36].

Romania-Specific Data

In Romania, the average maternal age is lower (27.1 years) compared to Western Europe (30–32 years) [37]. Having one of the highest rates of adolescent pregnancies in the European Union, some studies found that teenage mothers in rural Romania also face higher EOS risks. Limited prenatal care among younger age groups contributes to undetected infections during pregnancy, increasing neonatal vulnerability [20,38].

Romania has one of the highest rates of teenage pregnancies in Europe, as reported by UNICEF and European health agencies: in 2021, 10% of all births in Romania were to underage mothers (under 18 years old).

Romania has the second-highest rate of adolescent pregnancies (ages 15–19) in the European Union, with around 40 live births per 1000 adolescent girls annually [39,40].

Approximately 12.3% of all births in Romania occur to mothers under the age of 20, compared to the EU average of 5% [41]. Adolescent mothers are often unprepared for the demands of pregnancy and childcare, leading to higher risks of preterm births, low-birth-weight infants, and maternal complications.

Conversely, pregnancies in women over the age of 35 are also increasing in urban areas, attributed to shifting career priorities and delayed childbirth. Romanian studies report an increase in EOS cases associated with maternal age ≥35, attributing this to a higher prevalence of comorbidities like gestational diabetes, hypertensive disorders, and prolonged labor [11,38].

#### 3.2.2. Parity

Parity, defined as the number of times a woman has given birth, affects maternal readiness for prenatal monitoring, adherence, and infection prevention.

Conversely, primiparous women (first pregnancies) face risks due to inadequate maternal education and lack of awareness about prenatal healthcare [21].

Eastern Europe Trends

The number of pregnancies or parity (nulliparous, multiparous, or grand multiparous) significantly influences maternal health outcomes. Studies in Eastern Europe show that women with higher parity (≥4 births) are at increased risk for complications such as uterine rupture, postpartum hemorrhage, and anemia due to repeated pregnancies. Conversely, nulliparous women (having their first pregnancy) often lack the experience or knowledge to navigate healthcare access, leading to gaps in prenatal care. Berardi et al. (2017) emphasized that inadequate education about GBS screening is a common finding among first-time mothers, particularly in socioeconomically disadvantaged regions [25,30].

Romania-Specific Data

In Romania, a divide between urban and rural settings influences parity trends. Rural areas are characterized by higher birth rates and higher numbers of pregnancies per woman, partly due to limited contraceptive access and cultural attitudes favoring larger families.

Multiparous women (≥3 pregnancies) were identified as being at higher risk for unmonitored pregnancies and late prenatal care initiation in Romania, especially in rural areas, often attending fewer prenatal visits, assuming familiarity with pregnancy processes [14,15].

Grand multiparity (≥4 births) is disproportionately common among Roma women, who often face systemic barriers to family planning services. In urban Romania, fewer pregnancies and smaller family sizes are observed due to better access to contraception and changing societal norms, often reducing pregnancy-related risks when paired with quality prenatal care [5,36].

#### 3.2.3. Marital Status

Married women are more likely to attend prenatal care appointments than unmarried women. In Romania, cultural and financial support often correlates with marital status, influencing prenatal care access. Single mothers, especially in rural areas, face additional barriers, including stigma and economic constraints [15].

Some authors and WHO highlight higher EOS risks among single mothers in Romania due to financial constraints and reduced access to continuous medical support.

Others further illustrate that social support networks provided within traditional households improve access to prenatal care and compliance with medical recommendations for EOS prevention in newborns [16,17,31].

Eastern Europe Trends

Marital status is a critical demographic factor affecting maternal health, influencing societal support, family stability, and access to healthcare. Married women often experience better maternal outcomes due to increased family support and financial stability. In contrast, single or unmarried mothers face higher risks of unfavorable outcomes due to financial insecurity and lack of access to stable healthcare, often facing social stigma [7,13].

Romania-Specific Data

The marital rate in Romania has decreased over the past decade, reflecting changing societal norms. Approximately 30% of Romanian children are born to unmarried mothers, higher than the EU average of 25%. In rural areas, traditional family structures often lead to earlier marriages and childbearing, while in urban areas, delayed marriage and childbirth are increasingly common [36].

Studies highlight higher EOS risks among single mothers in Romania due to financial constraints and reduced access to continuous medical support. Single mothers and unmarried women, particularly among marginalized populations such as the Roma community, face significant challenges, including stigma, limited economic resources, leading to higher rates of complications [5,16,17].

#### 3.2.4. Literacy and Health Literacy

Low literacy rates among rural populations in Romania worsen disparities in prenatal care. Health literacy, a subset of general literacy, is a critical determinant of prenatal care compliance. Maternal education level plays a substantial role in prenatal care engagement and monitoring for EOS risk factors.

Lower maternal education levels correlate with higher neonatal mortality due to EOS, as education impacts health literacy and adherence to prenatal care recommendations. Studies from Eastern Europe show that higher educational attainment in mothers leads to improved neonatal outcomes, as these mothers are more likely to seek regular prenatal care and follow medical advice.

Eastern Europe Trends

Literacy rates and educational attainment are linked to maternal health outcomes. Women with higher levels of education are more likely to access prenatal care, understand pregnancy complications, and adhere to medical advice. Conversely, low literacy rates and poor educational attainment are associated with limited maternal health knowledge, reduced care-seeking behavior, and higher neonatal mortality rates [8,22].

Romania-Specific Data

While Romania’s overall literacy rate is high (98%), educational inequality persists between urban and rural areas. Women in urban centers are more likely to attain higher education, which correlates with better health literacy and proactive pregnancy monitoring [24]. In contrast, rural regions, especially among the Roma population, experience significantly lower levels of education. Maternal awareness of proper neonatal hygiene and timely healthcare seeking is critical, yet rural and uneducated populations often rely on limited or inaccurate health information resources [21,42].

Women with secondary or incomplete primary education are less likely to attend the recommended 8–12 prenatal visits, particularly in rural regions. Studies report that in Romania, lower education levels correlate with reduced prenatal visit rates and lower adherence to recommended screening protocols, including GBS and TORCH infections [15,31].

It is estimated that 72% of Roma women in Romania have not completed secondary school, limiting their access to information on reproductive health and prenatal care [5]. This educational disparity contributes to poor maternal outcomes, as these women are less likely to attend the recommended prenatal visits or identify early warning signs during pregnancy.

Ethnic Hungarian women in Romania showed disparities in health literacy; language barriers and low educational attainment were key factors that hindered their ability to engage with healthcare providers and comprehend health information [22].

### 3.3. Costs and Burdens Associated with Prenatal Care

Western European countries provide comprehensive coverage for prenatal care through national health systems, significantly reducing out-of-pocket expenses. In contrast, Eastern European countries like Romania rely on mixed public–private healthcare systems, resulting in higher financial burdens for families [16].

In Romania, uninsured women and those in low-income groups face the highest economic burden. Many women opt to skip routine tests or ultrasounds due to cost constraints. This financial pressure disproportionately affects single mothers and women in rural areas [12].

#### 3.3.1. Direct Costs

Maternal healthcare in Romania poses significant financial and systemic challenges, heavily affecting both families and the healthcare system. Despite public healthcare covering basic prenatal services, families often bear the costs of added diagnostics and specialized treatment not included in public insurance. These out-of-pocket expenses vary widely, ranging from EUR 500 to EUR 1500 per pregnancy depending on the complexity and nature of care [35]. This financial burden disproportionately affects low-income families and marginalized populations, especially those in rural areas, who frequently forgo essential care as a result of cost barriers [33,43].

The cost of pregnancy monitoring in Romania includes the following:

Consultations: Each prenatal visit costs approximately EUR 30–50 in private settings.

Ultrasounds: Routine ultrasounds cost EUR 50–100 per session in private clinics. Public healthcare facilities often provide these services for free, but availability is limited.

Laboratory tests: Essential blood and bacteriological tests cost EUR 10–50 each, depending on the facility [14]

#### 3.3.2. Summary of Indirect Costs

- Lost Income Due to Complications: Women facing pregnancy complications may be unable to work, resulting in lost wages and financial instability for their families [13,17,22]
- Travel Expenses: Women, particularly in rural areas, incur additional costs for transportation to healthcare facilities, which can be significant if access to care is limited [15,22].
- Long-term Healthcare Costs: Managing complications arising from pregnancy disorders can lead to ongoing healthcare expenses that burden families financially over time [12].
- Economic Impact on Family Income: Reduced working hours while managing health issues can have significant effects on overall family income, contributing to financial strain [26].
- Maternal Healthcare Disparities: Economic implications arise from disparities in maternal healthcare, where under-resourced communities may face higher indirect costs due to inadequate access to care [27].
- Long-term Effects of Maternal Morbidity: Health issues can have lasting economic effects on families, affecting their financial stability and overall quality of life [26].
- Reduced Outreach Capacity: Funding limitations can hinder maternal outreach efforts, reducing the availability of educational and healthcare resources for expectant mothers [16].
- Impact on Labor Force Participation: The pandemic has exacerbated issues related to labor force participation for those needing care, as many individuals had to prioritize health over work [19].
- High Complication Rates in Underserved Communities: Economic impacts are more pronounced in underserved communities, where high rates of complications can lead to increased healthcare costs and loss of income [14].
- Long-term Implications for Children: Maternal health issues can have long-term implications for children’s futures, including potential impacts on their health and economic opportunities [28].
- Time Lost Navigating Care Pathways: The time spent by mothers navigating culturally relevant care pathways detracts from their ability to engage in work or other productive activities [21].

#### 3.3.3. Summary of Hidden Costs

- Transportation and Time Off Work: Additional expenses arise from traveling to healthcare facilities, coupled with the need for time off work for medical appointments, impacting financial stability [15].
- Childcare Costs: Families incur costs for childcare for siblings while attending prenatal appointments, adding to the overall financial burden [22]
- Missed Work Opportunities: Attending multiple healthcare appointments can lead to missed work opportunities, resulting in lost income and reduced productivity [36].
- Emotional and Psychological Burdens: Navigating the healthcare system creates significant emotional and psychological stress for expectant mothers, complicating their overall health experience [26].
- Reduced Mental Health Support: Emotional distress can limit access to mental health resources vital for coping with the challenges of pregnancy and motherhood [12].
- Inadequate Follow-Up Care: Costs associated with insufficient follow-up care can lead to complications and additional healthcare needs, straining family resources [17].
- Emergency Obstetric Care Variability: The unpredictability of costs related to emergency obstetric care can create financial uncertainty for families [35].
- Unfunded Education Costs: Educational programs on prenatal care may lack funding, leading to missed opportunities for women to gain crucial knowledge about their health [26].
- Policy Implementation Challenges: Burdens arise when policies are implemented without adequate resources, affecting the quality and accessibility of maternal healthcare services [14].
- Pandemic-Related Psychological Toll: Fear stemming from the pandemic can deter individuals from seeking necessary healthcare, impacting maternal and child health outcomes [19].
- Transportation Resource Allocation: Time and resources spent on seeking transportation to care centers can detract from other essential activities, such as work or family care [21].
- Societal Costs for Marginalized Groups: Poorer health outcomes for marginalized groups lead to societal costs, as these populations may require more extensive healthcare services and support [27].
- Cultural Norms Impacting Decisions: Cultural norms can influence healthcare decisions, potentially leading to delays in seeking care or opting for less effective treatments [22,28].

## 4. Discussion

### 4.1. Comparison with Data Found in Our Study

#### 4.1.1. Maternal Age

The average maternal age found in the study was 27.6 years, closely aligned with the reported age for Romania, indicating that the sample is representative of national demographic trends. Our study found that 11.3% of the mothers were adolescent and 7.9% were underage, both numbers being lower than the national average, suggesting that our sample may have a slightly higher representation of mothers with better healthcare access and educational opportunities, which often correlate with delayed childbirth.

**Figure 2:**
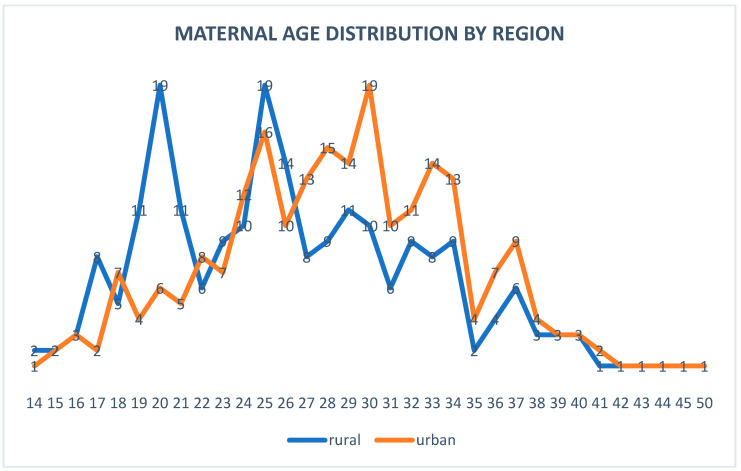
Graphic representation of maternal age distribution by region.

When analyzing maternal age by region, we found that urban areas have a higher proportion of mothers aged ≥ 35 compared to rural areas (8.4% versus 5.4%), consistent with European trends of delayed childbirth in urban regions, while adolescent pregnancies (<20 years) are significantly higher in rural areas (7.1% versus 4.3% in urban settings), as seen in Figure 2. Advanced maternal age (≥35 years) trends in urban areas also reflect the increased prioritization of careers and delayed childbirth, as mentioned for Europe. The data align with the Romanian context where adolescent pregnancies are more prevalent in rural areas due to limited education and family planning services.

#### 4.1.2. Parity

This study also examined parity, finding an average of 1.66 children per mother, which aligns closely with urban European and Romanian norms, where smaller families are the trend. This suggests a considerable portion of the study population either resides in urban areas or shows urban-like behavior about family size. The low prevalence of very high parity (≥4 children) as a dominant trend indicates limited representation of highly marginalized or rural populations with traditional family norms.

Rural areas show higher rates of parity (42.8% of the mothers had 2–3 children), with some cases of grand multiparity (≥4 children—7.5%), consistent with Romania’s rural norms favoring larger families, a trend influenced by traditional family values and limited contraceptive access, also mirroring European trends.

**Figure 3:**
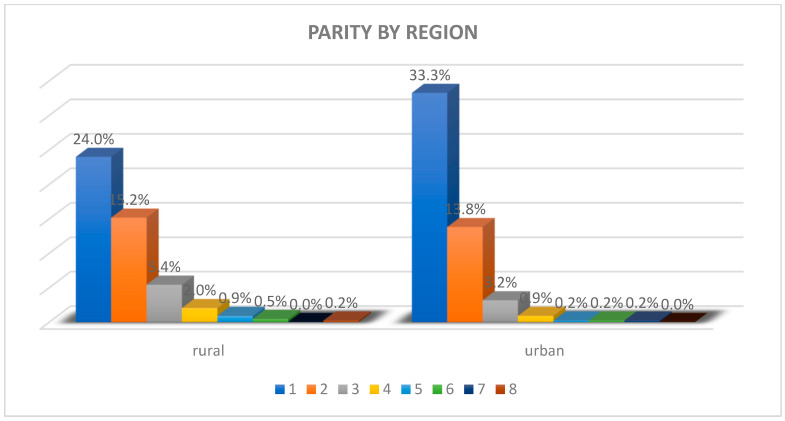
Graphic representation of parity by region.

Meanwhile, urban areas feature lower parity, with most mothers having only one (64.2%) or two children (26.6%), reflecting modern societal norms where financial considerations, career priorities, and lifestyle preferences are key contributors, as seen in Figure 3.

#### 4.1.3. Literacy

**Figure 4:**
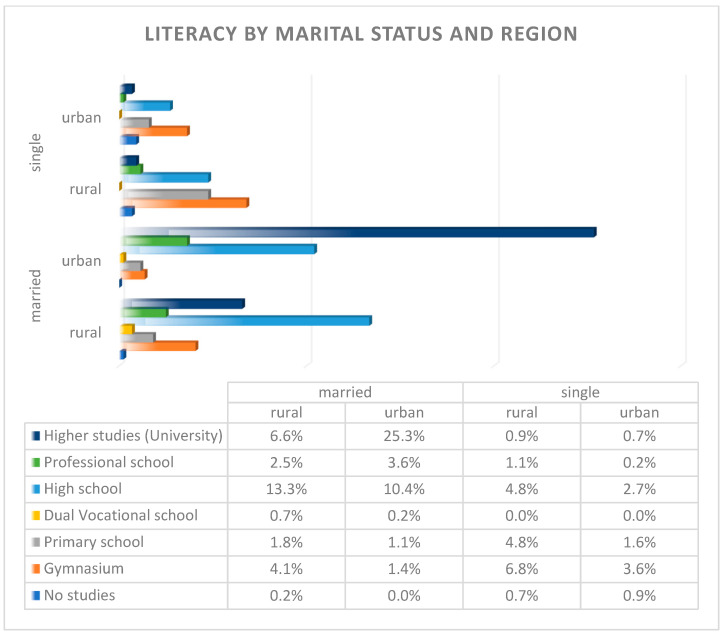
Graphic representation of maternal literacy level by marital status and region.

Our study reflects Romania’s educational disparities, particularly among marginalized groups like the Roma community in rural areas. The average level of education among the study population corresponds to high school education, but disparities were evident between rural and urban mothers, as represented in Figure 4.

Rural areas had a higher proportion of women with only primary- or gymnasium-level education, which strongly correlated with lower prenatal care utilization. Among those with lower education levels, a concerning 53.7% received incomplete prenatal care, while 4.9% had no prenatal care at all. These figures highlight the barriers that low educational attainment poses to accessing healthcare, a trend widely recognized in maternal health research across Europe.

Urban mothers dominate higher education levels (post-secondary and university degrees). Most mothers had high education levels and 90.9% received regular prenatal care, while only 9.1% had incomplete care, and none were entirely without care, highlighting the positive impact of higher education on healthcare engagement. This reinforces the well-established relationship between higher literacy levels and improved healthcare access, a phenomenon also observed in broader European studies that link maternal education with better neonatal outcomes.

#### 4.1.4. Marital Status

In Romania, about 30% of children are born to unmarried mothers, which aligns closely with this study’s finding of 28.7% unmarried mothers. European trends highlight better healthcare access for married women, supported by family stability, which is reflected in this study, where 82.7% of the married mothers received regular prenatal care.

Rural areas have a higher proportion of unmarried mothers (39.4%) compared to urban areas (18.8%), while urban areas show a larger percentage of married mothers (81.2%).

**Figure 5:**
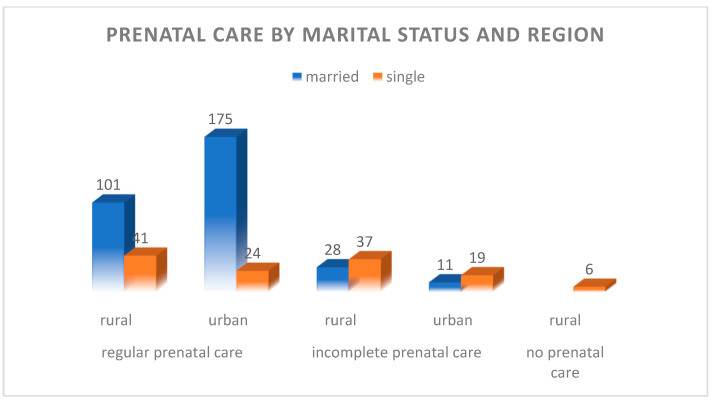
Graphic representation of prenatal care by marital status and region.

Married mothers in this study were significantly more likely to receive regular prenatal care (87.6%) compared to unmarried mothers (51.2%), reflecting Romanian and European trends, as represented in Figure 5.

These data match Romania’s rural trends where single mothers face socioeconomic challenges, affecting healthcare access (51.1% of these had incomplete or no prenatal care). At the same time, they are consistent with European observations of social stigma and financial constraints affecting single mothers, especially among marginalized groups.

### 4.2. Challenges in Prenatal Care in Romania

#### 4.2.1. Rural–Urban Divide

The rural–urban divide in maternal healthcare significantly affects outcomes for expectant mothers, particularly in Eastern Europe. Women living in rural areas often face considerable challenges in accessing high-quality prenatal and postnatal care, leading to disparities in health outcomes compared to their urban counterparts. Studies highlight that rural residents often encounter barriers such as limited healthcare facilities, longer travel distances, and fewer healthcare providers, which directly affect their ability to receive prompt care. Moreover, some emphasize that transportation costs can add a considerable financial burden for rural women, who may have to travel significant distances to reach healthcare centers. These access issues are exacerbated by insufficient public transport services and economic constraints, leading to increased missed appointments and inadequate prenatal care [17,23,44].

Studies illustrate that the health literacy level among rural women is often lower due to limited educational resources, which further complicates their ability to navigate the healthcare system [13,22]. This lack of awareness can lead to delayed health-seeking behaviors and increased risks for maternal and infant morbidity. In contrast, urban areas typically offer more comprehensive healthcare services, higher availability of specialists, and better educational resources, contributing to improved maternal health outcomes [36,44]. Additionally, the World Health Organization (2022) indicates that the concentration of healthcare resources in urban areas fosters innovation and better service delivery models, which rural regions can lack [8]. Women in rural areas often rely on general practitioners with limited obstetric ability. Addressing these disparities requires targeted investments in rural healthcare infrastructure and initiatives focused on improving access and education for expectant mothers in less populated areas [17].

Furthermore, the economic implications of the rural–urban divide are significant, as families in rural areas may struggle with the dual burden of healthcare costs and lost income due to complications arising from inadequate prenatal care [12]. This situation creates a cycle of disadvantage that affects maternal and child health outcomes. In response, policies must not only address healthcare shortages but also improve transportation and educational outreach programs aimed at rural residents, ensuring fair access to quality maternal care for all women, regardless of their geographical location.

#### 4.2.2. Cultural Barriers

Cultural barriers significantly affect the use of maternal healthcare services and can often lead to suboptimal health outcomes for women. In many communities, traditional beliefs and practices around pregnancy and childbirth influence women’s healthcare choices, which may prioritize traditional methods over modern medical interventions. Certain studies prove that cultural norms can lead to reluctance in seeking care outside the community, as women may feel that their needs are better addressed through traditional support systems. This cultural resistance can hinder women’s willingness to access necessary prenatal services, resulting in delays in the diagnosis and treatment of potentially serious complications [27].

Moreover, emotional factors intertwined with cultural beliefs often contribute to the stigma surrounding maternal health issues. Some note that women in certain cultures may experience feelings of shame or inadequacy when seeking clinical help, particularly for mental health concerns, further deterring them from utilizing available resources [28]. As some authors point out, this situation is compounded by the systemic lack of culturally competent care within healthcare facilities, as clinicians may not fully understand or respect the diverse cultural backgrounds of their patients. This disconnect can have serious consequences, leading to distrust in the healthcare system and further perpetuating health inequalities [26].

Addressing cultural barriers in maternal healthcare requires a multifaceted approach, incorporating culturally sensitive training for healthcare providers, community outreach programs, and partnerships with local leaders to promote awareness of the importance of maternal health services. As exemplified by Almeida et al. (2019), effective interventions need to consider the cultural contexts of the populations they serve, empowering women with knowledge and fostering trust in the healthcare system [21,45]. By addressing these cultural factors and engaging women meaningfully, healthcare providers can create pathways for improved maternal health outcomes and promote more inclusive healthcare practices that resonate with the values and beliefs of diverse communities [46,47,48].

#### 4.2.3. Policy and Infrastructure Gaps

Romania’s healthcare infrastructure struggles to meet the demand for prenatal care, particularly in underserved areas. Policymakers have introduced programs to improve maternal health, but implementation remains inconsistent due to funding and logistical challenges [16].

Policy and infrastructure gaps significantly hinder the provision of quality maternal healthcare services in various countries, particularly in Eastern Europe. One study asserts that inadequate healthcare policies often lead to insufficient funding and resource allocation, directly affecting the availability and quality of services offered to expectant mothers [26,49]. The inequities between urban and rural healthcare infrastructures further exacerbate these challenges, as rural areas frequently lack essential medical facilities and trained personnel [25]. Moreover, the disparities in healthcare resources create ongoing barriers to access, compelling women in underserved communities to travel long distances for critical prenatal and postnatal care [12]

The urgency of filling these gaps was particularly evident during the COVID-19 pandemic, which disrupted healthcare delivery and exposed the fragility of existing systems [26,50]. As noted in certain studies, the pandemic disproportionately affected vulnerable populations, highlighting the need for robust contingency planning within maternal healthcare frameworks to address sudden healthcare challenges. Policymakers must prioritize investments in healthcare infrastructure, ensuring that services are not only resilient but also equitably distributed across regions. This includes enhancing telehealth initiatives that can bridge gaps in service delivery for women in rural areas, as well as strengthening partnerships with community organizations to enhance outreach and education [13,22,51,52].

Furthermore, addressing policy gaps is crucial for creating a comprehensive maternal healthcare framework that encompasses preventive care, education, and mental health resources [3]. An integrated approach is needed, one that integrates maternal health services within broader public health initiatives and encourages cross-sector collaboration to create supportive environments for expectant mothers. This approach requires the development of policies that reflect the diverse needs of women, ensuring access to a continuum of care that ultimately improves health outcomes for mothers and their children [15]. By tackling these infrastructure and policy gaps, healthcare systems can move towards achieving fair and high-quality maternal healthcare for all women [53].

### 4.3. Recommendations for Improving Prenatal Care in Romania

#### 4.3.1. Enhance Access to Healthcare Services

To bridge the rural–urban divide in maternal healthcare access, it is crucial to improve the availability of healthcare facilities and professionals, particularly in rural areas. Policymakers should invest in building new clinics and enhancing existing healthcare infrastructure to ensure that all women, regardless of their geographical location, can access comprehensive prenatal care. Mobile health units could also be implemented to reach remote communities, providing essential services directly at the doorsteps of those in need [13,17,54,55].

#### 4.3.2. Improve Transportation and Support Services

Addressing transportation challenges is essential for increasing access to prenatal care. Government initiatives should focus on subsidizing transport costs for expectant mothers who need to travel long distances to healthcare facilities. Additionally, setting up partnerships with local transport services can ease access to healthcare appointments. Furthermore, implementing logistics support systems to coordinate transportation for vulnerable populations can significantly alleviate barriers to care [12,13,15].

#### 4.3.3. Increase Education and Awareness Campaigns

Increasing health literacy among expectant mothers is vital for improving prenatal care use. Community health education programs should be developed that focus on maternal health topics, including the importance of regular prenatal visits, recognizing warning signs during pregnancy, and understanding available resources. These educational efforts should be culturally sensitive and tailored to address the specific needs of different communities [13,21,35].

#### 4.3.4. Integrate Culturally Competent Care

Training healthcare providers in cultural competence is essential to address the diverse needs of Romania’s population effectively. Providers should be educated about the cultural beliefs and practices that influence their patients’ healthcare decisions, which can help build trust and encourage women to seek care. Collaborating with local community leaders and organizations can facilitate this process, ensuring that maternal health services are relevant and respectful of cultural norms [22,26,27].

#### 4.3.5. Strengthen Mental Health Services

Maternal mental health is a critical part of overall prenatal care. Policymakers should prioritize the integration of mental health support within prenatal care services, creating referral systems for women experiencing emotional distress. Training healthcare providers to recognize and address mental health issues can significantly improve maternal health outcomes and promote wellbeing during pregnancy [14,51].

#### 4.3.6. Improve Policy and Funding Allocation

It is imperative to conduct comprehensive reviews of current maternal health policies and practices to identify gaps in funding and services [36,56]. Increasing investment in maternal healthcare initiatives and expanding funding for prenatal programs can enhance accessibility and quality. Policymakers should use data from health assessments to inform decisions and prioritize areas most in need of improvement.

#### 4.3.7. Foster Partnerships and Community Involvement

Building partnerships between healthcare providers, community organizations, and local governments can facilitate the development of holistic maternal health services. Involving community stakeholders in the design and implementation of programs fosters a sense of ownership and ensures that services are tailored to meet local needs [19]. Engaging communities in outreach efforts can improve attendance at prenatal appointments and increase awareness of available resources.

## 5. Conclusions

Our study highlights maternal demographics and prenatal care disparities in Romania within a broader European context. Findings reveal significant rural–urban gaps in healthcare access, education, and socioeconomic conditions, with maternal age, parity, literacy, and marital status influencing neonatal outcomes [57]. EOS rates varied across studies due to differences in healthcare access, emphasizing systemic inequalities. While 82% of the studies demonstrated high quality with minimal bias, future research should prioritize larger sample sizes, transparent funding, and comparative designs for improved reliability [58,59].

Improving maternal healthcare in Romania requires enhanced access, education, and culturally competent care. Expanding rural prenatal services through mobile units and telemedicine can help bridge gaps, while integrating health literacy programs into communities can improve maternal education. Targeted interventions for marginalized groups, particularly Roma communities, remain essential. Subsidizing GBS and TORCH screenings would enhance early EOS detection and improve neonatal health [60].

Policy interventions should address healthcare infrastructure, transportation, and public insurance coverage for screenings. Strengthening adherence to WHO’s eight-visit prenatal care standard and introducing financial incentives could improve maternal healthcare participation. Transparency in research funding is also vital for informed policy decisions [61].

Future research should focus on regional variations in maternal healthcare, EOS risk factors, and long-term neonatal outcomes. Investigating telemedicine’s impact on prenatal care in underserved areas is crucial. Achieving WHO’s goal of reducing neonatal mortality below 12 per 1000 live births by 2030 requires Romania to focus on infection prevention, policy reform, and improved prenatal care to ensure healthier maternal and neonatal outcomes [62].

## Figures and Tables

**Table 1:** Review findings.

Study	Country	Maternal Age	Parity	Literacy	Marital Status
Panaitescu, A.M. et al. (2020) [11]	Romania	The study discusses the higher prevalence of hypertensive disorders in women aged ≥ 35, reflecting trends of advanced maternal age	Highlights increased risk for hypertensive disorders in nulliparous women compared to multiparous ones	Not discussed	Not discussed
Cobzeanu, M.L. et al. (2022) [12]	North-East Romania	Increased prevalence of accreta spectrum disorders in women above 30 years	Higher incidence in grand multiparous women, often associated with a history of cesarean delivery	The paper alludes to low health literacy rates contributing to delayed presentations and diagnosis of complications	Not discussed
Stativa, E. et al. (2014) [13]	Romania	Younger mothers were found to underutilize prenatal care services more than older mothers. Teenage pregnancies contributed to increased risk of complications.	Higher parity correlated with decreased use of prenatal care, particularly in rural and socioeconomically disadvantaged areas. First-time mothers were more likely to seek prenatal care.	Health literacy was closely tied to socioeconomic status and geographic location; women with lower literacy or educational attainment had higher rates of underutilization.	Unmarried women or single mothers were less likely to access adequate prenatal care. Cultural and social stigmas, along with economic hardships, create additional barriers for unmarried women.
Chanturidze, T. (2012) [14]	Romania	Trends of increasing maternal age, with a notable number of pregnancies occurring among women aged 30 and above, reflecting a shift towards delayed childbirth in urban areas	Women in rural areas show higher parity rates compared to urban populations due to limited access to contraception	Highlights disparities in health literacy; lower education levels are prevalent among rural populations, hindering understanding of maternal health	Observes a significant proportion of births occurring among unmarried women, particularly in younger women and marginalized groups.
Miteniece, E. (2021) [15]	Eastern Europe	Notes trends of adolescent pregnancies in rural Eastern Europe and delayed pregnancies beyond 30 years in urban areas	Higher parity in rural regions due to low contraceptive use	Low educational attainment linked to reduced prenatal care adherence, particularly in rural communities	High rates of pregnancies among unmarried women in marginalized ethnic groups and conservative communities
WHO (2004) [16]	Romania	Adolescent pregnancies remain common in rural areas due to limited access to education and contraception	Observes unintended pregnancies contributing to higher parity in underserved communities	Not discussed	Discusses the stigma surrounding unmarried pregnant women and its impact on healthcare access
Panaitescu, A.M. (2020) [17]	Romania	Adolescent pregnancies cited as a key issue in disadvantaged areas	Notes a higher birth rate among women with limited education	Links low literacy to disparities in access to prenatal care, particularly in rural Romania	Not available
Horga, M. (2004) [18]	Romania	Adolescents represent a significant percentage of mothers in rural areas	Low contraception use leads to higher parity in underserved populations	Not discussed	High proportion of pregnancies among unmarried women in disadvantaged communities
Cionca, O. et al. (2015) [19]	Romania	Emphasizes the high prevalence of teenage pregnancies in marginalized groups	Not discussed	Vulnerable populations, such as Roma women, show lower literacy levels, reducing engagement in prenatal care	Limited social support for unmarried women affects care access
Berardi, A. et al. (2017) [20]	Various European countries	The study identifies an increasing trend of pregnancies among older women (≥35 years) which is more pronounced in Western and Central Europe compared to Eastern Europe	Discusses variations in parity, noting that certain Eastern European countries have higher rates of multiparity compared to Western counterparts due to cultural norms and family planning policies	Mentions that mothers with higher literacy levels tend to utilize antenatal services more effectively and adhere to recommended healthcare guidelines	The article notes that marital status significantly impacts access to care; unmarried mothers in Eastern Europe often encounter stigmas that affect their healthcare utilization
Almeida, A.C. et al. (2019) [21]	Europe	The findings indicate a demographic shift with increasing maternal age at first childbirth across several Eastern European countries, mirroring trends observed in Romania	Reports a trend of high-parity pregnancies in Eastern European countries due to less access to effective family planning methods	The study emphasizes that lower levels of maternal education correlate with poorer outcomes in maternity care practices, particularly in Eastern Europe	Highlights that increased rate of births outside marriage, especially in Eastern Europe, influence maternal care-seeking behaviors and health outcomes
Sántha, Á. (2021) [22]	Romania, Hungary, Slovakia (focus on ethnic Hungarian mothers)	Younger mothers often struggle with understanding health information, resulting in less effective prenatal and postnatal care utilization	Women with higher parity had lower health literacy levels	Low levels of health literacy associated with lower educational attainment	Married women were more likely to have better health literacy and access to prenatal care

**Table 2:** Review findings (costs).

Study	Direct Costs	Indirect Costs	Hidden Costs	Burdens
Panaitescu, A.M. et al. (2020) [11]	Hospitalization costs due to hypertensive disorders, routine prenatal visits, and prescribed medications	Lost income for women unable to work due to complications	Additional expenses for transportation to healthcare facilities and time off work for appointments	Increased healthcare utilization leading to financial strain on families and the public health system
Miteniece, E. (2021) [23]	Out-of-pocket expenses for prenatal care services and consultations	Travel expenses for reaching healthcare centers, particularly in rural areas	Cost of childcare for siblings while attending prenatal appointments	Accessibility issues due to geographic location, leading to delayed or inadequate care during pregnancy
Stativa, E. et al. (2014) [13]	Lack of insurance and limited healthcare coverage; costs of medical consultations, laboratory tests and medications are significant for low-income families	Loss of income due to time off work and transportation costs for prenatal appointments for those living far from healthcare facilities	Informal payments to healthcare providers	Emotional and psychological stress due to limited access to care and financial costs.
Almeida, A.C. et al. (2019) [21]	Differences in healthcare expenditures for prenatal care across countries	Variation in lost productivity based on care accessibility	Emotional and psychological burdens of navigating the healthcare system	Disparities in care quality and access across different socioeconomic backgrounds
Cobzeanu, M.L. et al. (2022) [12]	Surgical costs associated with treating accreta spectrum disorders	Long-term healthcare costs for managing complications arising from these disorders	Emotional distress leading to reduced mental health support accessibility	Increased complexity in prenatal monitoring and treatment requiring specialized healthcare
Chanturidze, T. (2012) [14]	Funding for maternal health project initiatives and program management	Economic impact on family income due to reduced working hours while managing health issues	Costs associated with inadequate follow-up care	Systemic issues within healthcare infrastructure affecting maternal outcomes
Eurostat. (2023) [24]	Reported costs of maternal healthcare services across Europe, including Romania	Economic implications associated with maternal healthcare disparities	Variability in costs related to emergency obstetric care	Reflection of unequal access to care and resources across different regions
World Health Organization. (2022) [25]	Implementation costs for following WHO antenatal care guidelines	Long-term economic effects of maternal morbidity on families	Costs related to education on prenatal care that potentially go unfunded	Global disparities in adherence to recommended practices impacting maternal health
Horga, M. (2004) [18]	Resources dedicated to maternal care policies and programs	Reduced capacity for maternal outreach due to funding limitations	Burdens related to policy implementation without adequate resources	Inequities in maternal care access due to governmental resource allocation
Filip, R. et al. (2022) [26]	Increased expenses related to disruptions in care due to COVID-19	Impact on labor force participation for those needing care during the pandemic	Psychological toll from pandemic-related fear affecting health-seeking behavior	The strain on healthcare systems leading to postponement of necessary prenatal care
Cionca, O. et al. (2015) [19]	Costs associated with providing care to vulnerable populations	Economic impact from high rates of complications in underserved communities	Time and resources spent seeking transportation to care centers	Vulnerability affecting access to timely care, leading to poorer maternal health outcomes
Walsh, J. et al. (2011) [27]	Documentation of varying healthcare costs based on ethnic backgrounds	Long-term implications of maternal health issues on children’s futures	Societal costs associated with poorer outcomes for marginalized groups	Disparities in outcomes due to ethnic variations in healthcare access and quality
Pop, C.A. (2022) [28]	Expenses related to cultural sensitivity training for healthcare providers	Time lost for mothers navigating culturally relevant care pathways	Impact of cultural norms on healthcare decisions	Cultural and systemic barriers creating additional stress for expectant mothers
Sántha, Á. (2021) [22]	Paying for health services (consultations, medications, diagnostic tests); lack of resources for minority populations	Transportation expenses for those living in rural areas; time off work affecting household income, especially for single mothers	Language barriers that required hiring interpreters; informal payments to healthcare providers	Navigating socioeconomic challenges and ethnic discrimination.

## Data Availability

Data supporting reported results can be found at mirela.siminel@umfcv.ro; anca.vulcanescu@umfcv.ro, due to the need to uphold intellectual property rights and confidentiality agreements, ensure the integrity and accuracy of ongoing analyses, and comply with ethical and regulatory guidelines that govern data dissemination before formal publication.

## Supplementary Materials

The following supporting information can be downloaded at: https://www.mdpi.com/article/10.3390/children12030354/s1, Supplementary Material S1: PRISMA_2020_checklist; Supplementary Material S2: Risk of bias assessment. Supplementary Material S3: Extended search strategy. Reference [63] is cited in the supplementary materials.
