# Supplementary material for: Neonatal Mortality Due to Early-Onset Sepsis in Eastern Europe: A Review of Current Monitoring Protocols During Pregnancy and Maternal Demographics in Eastern Europe, with an Emphasis on Romania—Comparison with Data Extracted from a Secondary Center in Southern Romania
Source: Children (Basel). 2025 Mar 13;12(3):354. doi: 10.3390/children12030354 (PMC11941689; doi:10.3390/children12030354)
Supplement: Supplementary file 1 [file children-12-00354-s001.zip › Supplementary Material S2. Risk of bias.pdf]

Supplementary Material S2. Risk of Bias Assessment

Author(s)	Study Design Bias	Sample Size Bias	Outcome Reporting Bias	Funding Bias	Conflict of Interest Bias	Overall Bias
Panaitescu et al. (2020)	Medium	High	Medium	Low	Low	Medium
Cobzeanu et al. (2022)	Low	Medium	High	Medium	Low	Medium
Stativa et al. (2014)	Medium	Medium	Medium	Low	Low	Medium
Chanturidze (2012)	Low	Medium	Medium	Low	Low	Medium
Miteniece (2021)	Medium	Medium	High	Low	Low	Medium
WHO (2004)	Low	Low	Low	Low	Low	Low
Horga (2004)	Medium	Medium	Medium	Low	Low	Medium
Cionca et al. (2015)	Medium	High	Medium	Medium	Low	Medium
Berardi et al. (2017)	Low	Medium	High	Low	Low	Medium
Almeida et al. (2019)	Medium	Medium	Medium	Low	Low	Medium
Santha (2021)	Medium	Medium	Medium	Low	Low	Medium
Eurostat (2023)	Low	Low	Low	Low	Low	Low
WHO (2022)	Low	Low	Low	Low	Low	Low
Filip et al. (2022)	Medium	High	Medium	Medium	Medium	Medium
Walsh et al. (2011)	High	Medium	Medium	Low	Low	Medium
Pop (2022)	Medium	Medium	High	Medium	Low	Medium
Panaitescu et al. (2020)	Medium	High	Medium	Low	Low	Medium
Almeida et al. (2019)	Low	Medium	Medium	Low	Low	Low
Chanturidze (2012)	Medium	Medium	High	Low	Low	Medium
Cobzeanu et al. (2022)	Low	Medium	Medium	Medium	Low	Medium
WHO (2004)	Low	Low	Low	Low	Low	Low
Berardi et al. (2017)	Medium	Medium	High	Low	Low	Medium
Filip et al. (2022)	Medium	High	Medium	Medium	Low	Medium
Eurostat (2023)	Low	Low	Low	Low	Low	Low
Miteniece (2021)	Medium	Medium	Medium	Low	Low	Medium

1. Study Design Bias (15%): Bias introduced by the choice of study design, particularly in non-comparative or observational studies. Observational designs reduce generalizability and hinder establishing strong causal relationships.
Example: Studies like *Panaitescu et al. (2020)* relied on observational designs, limiting causative conclusions. Encourage randomized controlled trials (RCTs) or comparative cohort designs to improve causal inferences.

2. Sample Size Bias (20%): Bias from small or unrepresentative sample sizes, especially in niche populations. Small sample sizes can lead to underpowered studies, making results less reliable.
Example: *Cionca et al. (2015)* focused on rural and marginalized communities but with small sample sizes. Design studies with adequate sample sizes and ensure proportional representation across demographics

3. Outcome Reporting Bias (25%): Selective reporting of positive or specific outcomes while ignoring other important results. Skewed reporting limits the comprehensiveness of findings and may mislead policymakers.
Example: *Berardi et al. (2017)* focused solely on infection outcomes without considering broader maternal impacts. Promote transparent and standardized reporting across all predefined study outcomes.

4. Funding Bias (10%): Bias stemming from study sponsorship, often by organizations with vested interests. Lack of transparency erodes trust and may skew study priorities.
Example: Studies like *Cobzeanu et al. (2022)* lacked transparency in funding, raising questions about potential bias. Mandate full disclosure of funding sources and ensure studies are independent of sponsor influence.

5. Conflict of Interest Bias (5%): Bias introduced when researchers have personal or professional stakes in study outcomes. Potential conflicts may influence study objectives and data interpretation.
Example: Rarely reported explicitly but potentially present in pandemic-pandemic-focused studies like *Filip et al. (2022)*. Implement stricter declarations of conflicts of interest to uphold research integrity.

Overall Bias = 18%

Recommendations for Future Research
Improve Study Design: Use RCTs or comparative cohort studies to strengthen evidence quality.
Ensure Adequate Sample Sizes: Leverage national databases or collaborative research efforts to include diverse and larger sample sizes.
Standardize Reporting: Use guidelines like CONSORT or PRISMA for uniform and complete outcome reporting.
Enhance Funding Transparency: Mandate disclosure of funding sources and ensure independence from sponsors.
Address Conflicts of Interest: Strengthen oversight of conflicts by independent review boards.
Invest in Training and Resources: Train researchers in bias mitigation and incentivize quality, unbiased research
