# Supplementary material for: Neonatal Mortality Due to Early-Onset Sepsis in Eastern Europe: A Review of Current Monitoring Protocols During Pregnancy and Maternal Demographics in Eastern Europe, with an Emphasis on Romania—Comparison with Data Extracted from a Secondary Center in Southern Romania
Source: Children (Basel). 2025 Mar 13;12(3):354. doi: 10.3390/children12030354 (PMC11941689; doi:10.3390/children12030354)
Supplement: Supplementary file 1 [file children-12-00354-s001.zip › Supplementary Material S3 - Extended search strategy.pdf]

Supplementary Material S3. **Extended Search Strategy**

Objective: To systematically identify peer-reviewed research and reports on neonatal mortality, early-onset sepsis (EOS), maternal health, and prenatal monitoring protocols in Eastern Europe, incorporating literature from reputable organizations such as UNICEF and WHO while adhering to strict inclusion and exclusion criteria.

PubMed

(("neonatal mortality" OR "perinatal mortality" OR "infant mortality") AND ("early-onset sepsis" OR "neonatal sepsis" OR "perinatal infections") AND ("maternal health" OR "pregnancy outcomes" OR "perinatal care") AND ("prenatal monitoring" OR "antenatal screening" OR "maternal surveillance")
AND ("Eastern Europe" OR "Romania" OR "Poland" OR "Bulgaria" OR "Ukraine" OR "Hungary" OR "Czech Republic" OR "Slovakia" OR "Serbia" OR "Croatia")) AND (english[Language] AND 2000:2024[Date - Publication] AND journal article[Publication Type])

• Filters applied: English language, full-text availability, published between 2000-2024, human studies, peer-reviewed journals.

Google Scholar

("Neonatal mortality" OR "perinatal mortality") AND ("prenatal monitoring" OR "antenatal screening") AND ("maternal health") AND ("Eastern Europe" OR "Bulgaria" OR "Romania" OR "Poland" OR "Ukraine" OR "Hungary" OR "Czech Republic" OR "Slovakia" OR "Serbia" OR "Croatia")
AND ("early-onset sepsis" OR "neonatal sepsis") AND (after:2000 before:2024)

• Filters applied manually: Only peer-reviewed papers, full-text availability, English language.

Open Access Journals (DOAJ, PLOS ONE, BMC Pregnancy & Childbirth, The Lancet Global Health)

("Neonatal mortality" OR "Perinatal mortality") AND ("Prenatal monitoring" OR "Antenatal screening") AND ("Maternal health" OR "Perinatal care") AND ("Eastern Europe" OR "Bulgaria" OR "Romania" OR "Poland" OR "Ukraine" OR "Hungary" OR "Czech Republic" OR "Slovakia" OR "Serbia" OR "Croatia")AND ("Early-onset sepsis" OR "Neonatal sepsis") AND (2000-2024)4. WHO & UNICEF Reports Search Strategy:

WHO and UNICEF Official Websites

"Neonatal mortality" OR "Infant mortality" OR "Maternal health surveillance" AND "Eastern Europe" AND ("Reports" OR "Surveillance data" OR "Guidelines" OR "Epidemiological trends") AND (2000-2024)

WHO Database Filters Applied: Reports, guidelines, and epidemiological data from 2000-2024.

UNICEF Reports Reviewed: Global and regional data on neonatal mortality in Eastern Europe.

Inclusion and Exclusion Criteria

Inclusion Criteria:

- Topic relevance: Studies examining neonatal mortality, EOS, maternal health, or prenatal monitoring in Eastern Europe.
- Monitoring focus: Research addressing prenatal monitoring protocols or maternal demographics.
- Peer-reviewed journal articles and official reports from WHO, UNICEF, and Euro-Peristat.
- Timeframe: Studies and reports published between 2000 and 2024.
- Language: English only.
- Full-text availability.

Exclusion Criteria:

- Studies without full-text access.
- Non-research articles (e.g., opinion pieces, editorials, conference abstracts).
- Studies focused on non-human subjects.
- Reports focusing on regions outside Europe.
